# Probiotic Properties of *Bacillus* Strains Isolated from Stingless Bee (*Heterotrigona itama*) Honey Collected across Malaysia

**DOI:** 10.3390/ijerph17010278

**Published:** 2019-12-31

**Authors:** Fatin Aina Zulkhairi Amin, Suriana Sabri, Maznah Ismail, Kim Wei Chan, Norsharina Ismail, Norhaizan Mohd Esa, Mohd Azmi Mohd Lila, Norhasnida Zawawi

**Affiliations:** 1 Laboratory of Molecular Biomedicine, Institute of Bioscience, Universiti Putra Malaysia, Serdang 43400, Selangor, Malaysia; GS50839@student.upm.edu.my (F.A.Z.A.); maznahis@upm.edu.my (M.I.); chankim@upm.edu.my (K.W.C.); norsharina@upm.edu.my (N.I.); nhaizan@upm.edu.my (N.M.E.); 2 Enzyme and Microbial Technology Research Center, Faculty of Biotechnology and Biomolecular Sciences, Universiti Putra Malaysia, Serdang 43400, Selangor, Malaysia; suriana@upm.edu.my; 3 Department of Microbiology, Faculty of Biotechnology and Biomolecular Sciences, Universiti Putra Malaysia, Serdang 43400, Selangor, Malaysia; 4 Department of Nutrition and Dietetics, Faculty of Medicine and Health Sciences, Universiti Putra Malaysia, Serdang 43400, Selangor, Malaysia; 5 Department of Veterinary Pathology and Microbiology, Faculty of Veterinary Medicine, Universiti Putra Malaysia, Serdang 43400 UPM, Selangor, Malaysia; azmi@upm.edu.my; 6 Department of Food Science, Faculty of Food Science and Technology, Universiti Putra Malaysia, Serdang 43400, Selangor, Malaysia

**Keywords:** stingless bee honey, probiotic *Bacillus* strains, molecular identification, antimicrobial activity, pathogenic bacteria

## Abstract

This study aimed to isolate, identify, and evaluate the probiotic properties of *Bacillus* species from honey of the stingless bee *Heterotrigona itama*. *Bacillus* spp. were isolated from five different *H. itama* meliponicultures, and the isolates were characterized through Gram-staining and a catalase test. Tolerance to acidic conditions and bile salt (0.3%), hydrophobicity, and autoaggregation tests were performed to assess the probiotic properties of the selected isolates, *B. amyloliquefaciens* HTI-19 and *B. subtilis* HTI-23. Both *Bacillus* isolates exhibited excellent antimicrobial activity against both Gram-positive and Gram-negative bacteria and possessed significantly high survival rates in 0.3% bile solution for 3 h. Their survival rates in acidic conditions were also comparable to a commercial probiotic strain, *Lactobacillus rhamnosus* GG. Interestingly, the hydrophobicity and autoaggregation percentage showed no significant difference from *L. rhamnosus* GG, a commercial probiotic strain. The results from this study suggest that *B. amyloliquefaciens* HTI-19 and *B. subtilis* HTI-23 isolated from stingless bee honey have considerably good probiotic properties. Therefore, more studies should be done to investigate the effects of these bacteria cultures on gastrointestinal health.

## 1. Introduction

Stingless bee species are native to the tropics and subtropics of the world, including Australia, Africa, Southeast Asia, and parts of Mexico and Brazil. They are known for their role as important pollinators of both wild and cultivated flowering plants in different crops and orchards [1]. They produce honey, a natural sweet substance originating from nectar or blossoms that the bees collect, transform, and combine with specific substances of their own to ripen and mature [2]. Stingless bee honey is considered to be natural and organic food with high nutritional and therapeutic value [3]. It has significantly higher moisture content, water activity, ash content, and free acidity than does honeybee honey, while the pH and total soluble solid content are slightly lower [4]. Other than those components, proteins, amino acids, enzymes, organic acids, mineral elements, and vitamins are also present in honey, but only in small amounts [5,6]. These characteristics, especially lower reducing sugars and higher moisture content compared to the honey of honeybees, will eventually lead to the fermentation of stingless bee honey [7].

The fermentation of honey involves the presence of fungi (filamentous and yeast) and lactic acid bacteria, making stingless bee honey a plentiful source of microorganisms, with some of them exhibiting probiotic characteristics [8,9,10]. In addition, the humid and warm environment in a beehive provides optimum conditions for the growth of certain microbes. *Bacillus* spp. are Gram-positive and spore-former bacteria that can be widely found in soil and plants. However, due to their spore-forming characteristics, which are highly stable in acidic pH, they can also colonize different environments, such as honey and various food matrices [11]. For example, *Bacillus* species such as *B. subtilis*, *B. licheniformis*, *B. pumilus*, and *B. amyloliquefaciens* have been detected in the fermentation of both stingless bee honey and *Apis mellifera* honey [12,13]. These bacteria have advantages over non-spore formers such as lactic acid bacteria (LAB) due to their heat-resistant spores [14].

The identification of *Bacillus* species with antimicrobial properties might provide additional commercial value to stingless bee honey. The presence of *Bacillus* spp. in honey could originate from the bee itself or other environmental factors such as pollen, dust, and air [15]. *Bacillus* and many other genera, such as *Lactobacillus, Lactococcus, Bifidobacterium, Leuconostoc,* and *Pediococcus* are regarded as probiotics [16]. Probiotics are defined as “live microorganisms in which, when administered in adequate amounts, will confer a health benefit to the host” [17]. *B. subtilis* of different strains has been included as a dietary supplement in both human and animal diets through selected fermented foods such as natto, soybean, or any other probiotic supplement [18,19,20].

Most of the bacteria in the genus *Bacillus* are not harmful to mammalians, with the exception of *B. cereus and B. anthracis*. *Bacillus* spp. produce a vast variety of functional secondary metabolite-like antibiotics, bioinsecticides, enzymes, and lipopeptides, such as iturin, surfactin, fengycins, bacteriocins, and bacteriocin-like inhibitory substances (BLISes), which are also known as antimicrobial compounds. These biologically and commercially important characteristics make them a suitable candidate for uses as probiotic bacteria [11,21,22]. The significant role of probiotics is based on their antagonistic or antimicrobial activities against enteropathogenic bacteria. This is generally a result of bacteriocin secretion by the probiotic cultures or competitive metabolic interactions between probiotics and pathogens [23,24].

Most studies of the microorganisms associated with stingless bees have been carried out with the objective of describing the bacterial and fungal communities associated with these bees. While there have been extensive studies on LAB isolated from honeybees and stingless bee honey, little is known about the antimicrobial potential of *Bacillus* strains from the honey of *Heterotrigona itama* against some pathogenic bacteria. Herein, the study aimed to isolate, identify, and assess the probiotic properties of *Bacillus* species from the raw honey of *H. itama* from different meliponiculture places in Malaysia. To our knowledge, data on the strains and bioactivity of *Bacillus* species in raw stingless bee honey in Malaysia are still scarce, and therefore this study might provide some information on the probiotic properties of the nonpathogenic *Bacillus* strains isolated from the honey of *H. itama.*

## 2. Materials and Methods 

### 2.1. Honey Samples

Fifty milliliters of five raw honey samples were directly collected from the stingless beehives (*H. itama*) of different local apiarists located in Seri Kembangan (Selangor), Serdang (Selangor), Batang Benar (Negeri Sembilan), Segamat (Johor), and Sematan (Sarawak). Collected samples were stored in a sterile bottle at 4 °C before further analysis.

### 2.2. Isolation of Bacterial Strains from Stingless Bee Honey

Five-milliliter aliquots of stingless bee honey were added to 5 mL of nutrient broth (Oxoid, Basingstoke, UK) and incubated at 37 °C for 24 h. The culture was transferred to a 50-mL centrifuge tube and spun at 1500× *g* for 15 min, and then the supernatant was discarded. A total of 100 µl of 0.85% saline was added into the pellet and homogenized by vortexing for 10 sec. The mixture was then spread onto nutrient agar (Oxoid, Basingstoke, UK) through the spread-plate method. All experiments were done in triplicate. After being dried, the plates were incubated at 37 °C for 16–24 h. The bacterial isolates were streaked onto new plates to obtain a single colony. The colonies and microscopic morphologies were observed. A catalase test and Gram staining were performed according to Patel et al. [25].

### 2.3. Bacterial Strains and Growth Conditions

A total of 23 *Bacillus* strains isolated from stingless bee honey were included in this study. Pathogenic strains (*Escherichia coli, Salmonella thyphimurium, Klebsiella pneumonia, Pseudomonas aeruginosa*, and *Staphylococcus aureus*) were kindly supplied by the Enzyme and Microbial Technology Research Center, Faculty of Biotechnology and Biomolecular Sciences, Universiti Putra Malaysia, Malaysia. The strains were maintained at −80 °C in nutrient broth (Oxoid, Basingstoke, UK) with 20% (v/v) glycerol and were propagated three times in nutrient broth for activation prior to experimental use. 

### 2.4. Identification of Bacteria Using Molecular Technique

#### Genomic DNA Extraction, 16S rRNA Amplification, and Gene Analysis

Genomic DNA from pure cultures was extracted using a GF-1 Bacterial DNA Extraction Kit (Vivantis Technologies Sdn Bhd, Malaysia) according to the manufacturer’s instructions: 16S rRNA genes were amplified using a set of universal primers, 27F (5′-AGAGTTTGATCCTGGCTCAG-3′) and 1429R (5′-CGTTACCTTGTTACGACTT-3′) [26]. All PCR reactions were performed in 2X Taq Master Mix (Vivantis Technologies Sdn. Bhd., Malaysia) and amplified using a Thermal XP Cycler (BIOER Technology). PCR amplicons were sent for sequencing to MyTACG Bioscience Enterprise, Malaysia. The sequences obtained were analyzed using National Center of Biotechnology Information (NCBI) BLAST, and phylogenetic trees were constructed using MEGA7 software [27]. The following strains were used as a reference sequence for the phylogenetic analysis: *B. altitudinis* strain bacteria VII (KT427442), *B. pumilus* strain ML568 (KC692176), *B. pumilus* strain HB29 (KM659230), *B. subtilis* strain BSFLG01 (MF196314), *B. amyloliquefaciens* strain BA17 (MH891764), *B. amyloliquefaciens* strain 13 (HM107806), *B. megaterium* strain SX1 (MF431747), *B. aryabhattai* SX3 (MF431749), and *Salmonella enterica* spp. *enterica* strain LT2 as the outgroup.

### 2.5. Antimicrobial Activity Assessment

Antimicrobial activity was assessed using an agar well-diffusion method with slight modifications [28]. The turbidity of bacterial suspensions, adjusted to match the standard McFarland 0.5 (approximately 10^8^ colony forming unit, CFU/mL), was spread onto the plate. A 7-mm diameter well was punched aseptically onto the Mueller–Hinton agar (Oxoid, Basingstoke, UK) using the reverse end of a sterile 1-mL pipette tip. Tetracycline (20 µg/mL) was used as a positive control. A total of 100 µL of test agent was seeded into each well. A probiotic strain, *Lactobacillus rhamnosus* strain GG, was used as the reference strain. After incubation at 37 °C for 16–24 h, the diameter of the clear zone was measured. 

### 2.6. Screening for Probiotic Properties

#### 2.6.1. Acid and Bile Tolerance

Acid and bile tolerance were performed according to the method described by Klingberg et al. [29], with slight modifications. Bile tolerance was examined in nutrient broth (Oxoid, Basingstoke, UK) containing 0.3% (w/v) oxgall bile (Sigma-Aldrich, St. Louis, MO, USA). A volume of 100 μL of cell suspensions of *Bacillus* strains cultured for 18 h (approximately 10^7^ CFU/mL) were inoculated into nutrient broth (Oxoid, Basingstoke, UK) without bile and into nutrient broth (Oxoid, Basingstoke, UK) containing 0.3% (w/v) oxgall bile (Sigma-Aldrich, Missouri, US). The mixtures were incubated at 37 °C. Samples were taken at various times (0 h and 3 h), serially 10-fold-diluted using phosphate-buffered saline, PBS (pH 7.4), and plated in duplicate onto nutrient agar (Oxoid, Basingstoke, UK). The plates were incubated at 37 °C for 24 h. After the incubation period, viable bacterial colonies were counted and recorded. 

For acid tolerance, the isolates were incubated overnight in nutrient broth at 37 °C. Overnight cultures were harvested by centrifugation (1500× *g*, 4 °C, 20 min). Harvested cells were washed twice with phosphate-buffered saline (PBS) before being resuspended into nutrient broth (pH 7.0) which acts as control and nutrient broth (pH 2.0), adjusted with 0.1 M HCl. Samples were withdrawn after a time interval of 0 h and 3 h and were serially diluted in phosphate-buffered saline (PBS, pH 7.4) before being plated onto nutrient agar plates and incubated at 37 °C for 24 h. Cell viability was assessed by the plate count method, and the results are expressed as log CFU/mL. Both experiments were performed in triplicate.

The survival rate (SR) was calculated according to the equation below:

SR = (*N*1/*N*0 ×100%)

where *N*1 (log CFU/mL) is the total viable count of selected species after treatment (3 h), and *N*0 (log CFU/mL) represents the total viable count of selected species before treatment (0 h). A CFU is a colony-forming unit.

#### 2.6.2. Hydrophobicity

Bacterial adhesion was determined to assess the adherence potential of microorganisms to surface hydrocarbons, which is a measure of adhesion to epithelial cells of the gut. The hydrophobicity of the selected *Bacillus* isolates was measured according to the method of Kos et al. [30], with some modifications. Following overnight incubation, bacteria were harvested in the stationary phase by centrifugation at 1500× *g* for 15 min, washed once, and resuspended in phosphate-buffered saline (PBS), pH 7.4, to an absorbance (*A =* 600 nm) of about 0.25 ± 0.05 (*A_0_*) in order to standardize the number of bacteria (10^7^–10^8^ CFU/mL). Then, an equal volume of xylene (Fisher Scientific, Waltham, MA, USA) was added. After a 10-min preincubation at 37 °C, the cell suspensions were mixed well through vortexing for 2 min and were incubated at 37 °C for 1 h for aqueous and organic phase separation. The aqueous phase was carefully removed after incubation, and its absorbance was measured at 600 nm (*A_1_*). The percentage of bacterial adhesion to solvent was calculated as:

Auto-aggregation (%) = 1 − (A_1_/A_0_) × 100,

A_0_ = Absorbance at 0 h (600 nm),

A_1_ = Absorbance at 1 h (600 nm).

#### 2.6.3. Autoaggregation

Autoaggregation assays were performed according to Del Re et al. [31] with certain modifications. Bacteria were grown overnight at 37 °C in nutrient broth (Oxoid, Basingstoke, UK). The cells were harvested by centrifugation at 5000× *g* for 15 min and washed twice in phosphate-buffered saline (PBS). The initial concentration was adjusted to an optical density (OD) (*A* = 600 nm) of 0.25 ± 0.05 (*A_0_*) to give viable counts of approximately 10^8^ CFU/mL. Cell suspensions (4 mL) were mixed by vortexing for 10 s, and autoaggregation was determined over 24 h of incubation at 37 °C. In addition, 1 mL of the upper suspension was transferred to another tube, and the absorbance (*A*) was measured at 600 nm. The autoaggregation percentage is expressed as

Auto-aggregation (%) = 1 − (A_t_/A_0_) × 100,

A_0_ = Absorbance at 0 h (600 nm),

A_t_ = Absorbance at 24 h (600 nm).

### 2.7. Safety Assessment

#### 2.7.1. Antibiotic Susceptibility

The antibiotic susceptibility of the selected *Bacillus* strains was tested using a disk diffusion method according to Clinical and Laboratory Standard Institute (CLSI) performance standards for antimicrobial susceptibility testing [22]. Eleven kinds of antibiotics (Oxoid, Basingstoke, UK) were used: Ampicillin (AMP, 10 µg), Chloramphenicol (C, 30 µg), Ciprofloxacin (CIP, 5 µg), Erythromycin (E, 15 µg), Gentamycin (CN, 10 µg), Kanamycin (K, 30 µg), Tetracycline (TE, 30 µg), Teicoplanin (TEC, 30 µg), Vancomycin (VA, 30 µg), Rifampicin (RD, 30 µg), and Streptomycin (S, 10 µg). *Bacillus* cultures, adjusted to approximately 1 × 10^8^ CFU/mL using the 0.5 McFarland standard, were spread onto nutrient agar plates. Antibiotic discs were loaded onto the agar. The diameter of the inhibition zone for each antibiotic was detected after incubation at 37 °C for 24 h.

#### 2.7.2. Blood Hemolysis

The selected *Bacillus* strains were streaked on Columbia sheep blood agar containing 5% (w/v) sheep’s blood (Oxoid, Basingstoke, UK) and incubated at 37 °C for 24 h [32].

## 3. Results

### 3.1. Isolation and Preliminary Detection of Bacillus Isolates

Aerobic bacteria were isolated from all samples of stingless bee honey with varied concentrations: the mean values were between 9.7 × 10^0^ CFU/g and 3.67 × 10^2^ CFU/g. Out of 5 honey samples, a total of 58 isolates of different morphological characteristics were selected and identified as *Bacillus* species based on early morphological examination. The selected colonies appeared to be circular and creamy and were not pigmented.

The shapes of the colonies were examined on the plates after incubation periods of 24 h at 37 °C. The isolates were initially identified using morphological and biochemical tests. Microscopic characterization proved that 94% of them were Gram-positive and rod-shaped or also known as *Bacillus* (Table 1). The Gram-positive and catalase positive isolates were further tested for their tolerance of 7% NaCl, as this is one of the desirable technological properties of probiotic bacteria. Out of 58 isolates, only 23 of them were able to tolerate high concentrations of 7% NaCl (Table 1) and were therefore selected for further identification using 16S rRNA gene sequence analysis.

### 3.2. Molecular Identification through 16S rRNA Gene Sequence Analysis

**Figure 1:**
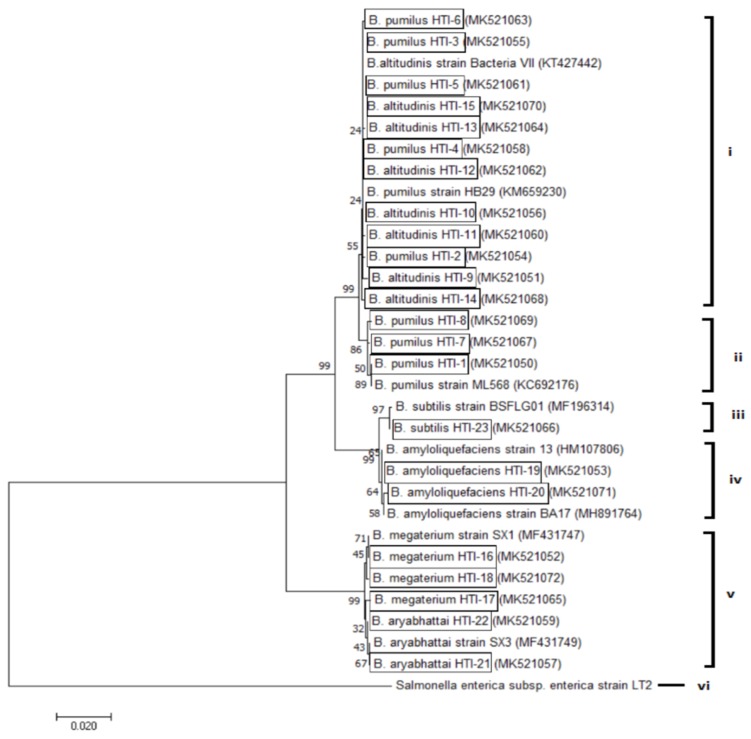
Evolutionary relationships of taxa. The evolutionary history was inferred using the neighbor-joining method. An optimal tree with the sum of branch lengths = 0.34080293 is shown. The percentage of replicate trees in which the associated taxa clustered together in the bootstrap test (1000 replicates) is shown next to the branches. The tree is drawn to scale, with branch lengths in the same units as those of the evolutionary distances used to infer the phylogenetic tree. The evolutionary distances were computed using the maximum composite likelihood method and are in the units of the number of base substitutions per site. The analysis involved 32 nucleotide sequences. All positions containing gaps and missing data were eliminated. There were a total of 1405 positions in the final dataset. Evolutionary analyses were conducted in MEGA7.

In general, a 16S rRNA gene sequence analysis of 23 selected isolates revealed that the dominant *Bacillus* species in this study were *B. pumilus* (34%) and *B. altitudinis* (33%), followed by *B. megaterium* (13%), *B. amyloliquefaciens* (8%), *B. aryabhattai* (8%), and *B. subtilis* (4%) (with 98%–100% similarities). Sequences of the 16S rRNA genes from the 23 new isolates of *Bacillus* were deposited in the GenBank, National Center of Biotechnology Information (NCBI) database. To further examine the phylogenetic affiliation, the 16S rRNA gene sequences of all isolates were aligned with eight closely related reference sequences (Figure 1). 

### 3.3. Distribution of the Bacillus Species Isolated from Stingless Bee Honey from Different Geographical Locations

**Figure 2:**
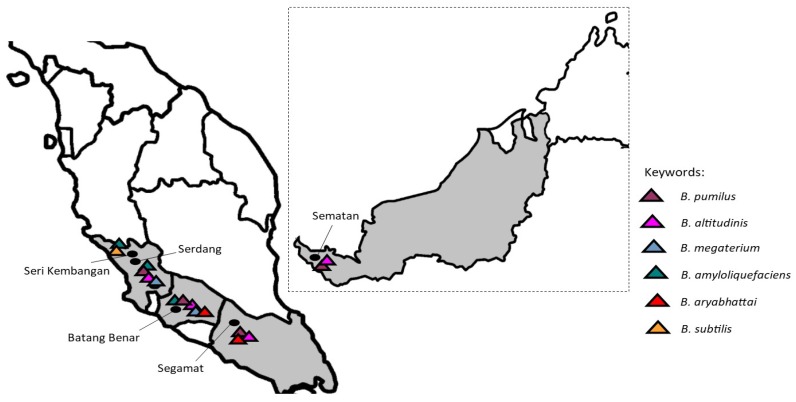
Distribution of the *Bacillus* species isolated from stingless bee (*Heterotrigona itama*) honey from different geographical locations.

In the results, the honey samples from Batang Benar, Negeri Sembilan, and Serdang (Selangor) showed more variation in their *Bacillus* species compared to the other samples. Even though the species of bacteria and the number of colonies differed between the sites sampled, *B. pumilus* and *B*. *altitudinis* were the most widely distributed, as they were detected in four out of five samples of raw *H. itama* honey collected from different geographical locations (Figure 2). Our results coincide with a study on Argentine honeys, where the presence of *B. pumilus,* together with *B. cereus* and *B. laterosporus*, was found among the 70 samples examined [33]. Recently, *Bacillus* spp. were also reported as the most frequently isolated bacteria in honey, making up 67% of total isolates [34]. 

### 3.4. Antimicrobial Test against Pathogenic Bacteria

**Figure 3:**
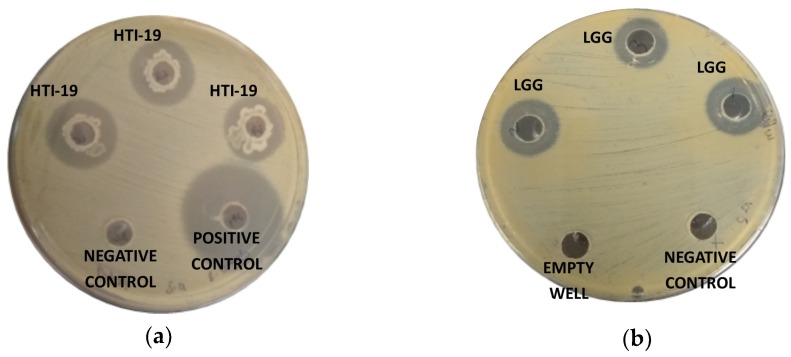
Inhibition zone of (**a**) potential probiotic *B. amyloliquefaciens* HTI-19 (HTI-19) against *Staphylococcus* *aureus* and (**b**) commercial probiotic *Lactobacillus rhamnosus* GG (LGG) against *S. aureus.*

The antagonistic activity of the isolates in this study was evaluated against Gram-positive and Gram-negative pathogenic bacteria: *S. aureus, B. cereus, S. thyphimurium, E. coli, K. pneumonia,* and *P. aeruginosa.* The results were compared to a commercial probiotic strain, *Lactobacillus rhamnosus* GG (Table 2). Nineteen isolates that were Gram-positive bacteria, rod-shaped, catalase-positive, and were able to grow in the presence of 7% NaCl were selected. These strains exhibited inhibitory effects against at least one of the tested pathogens, except for *B. pumilus* HTI-3, *B. megaterium* HTI-16, *B. megaterium* HTI-17, *B. megaterium* HTI-18, *B. aryabhattai* HTI-21, and *B. aryabhattai* HTI-22. Two isolates with the most excellent antagonistic activity against the tested bacteria were *B. amyloliquefaciens* HTI-19 and *B. subtilis* HTI-23, where the degree of inhibition spectrum of *B. amyloliquefaciens* HTI-19 were almost comparable to *L. rhamnosus* GG (Figure 3)). *B. amyloliquefaciens* HTI-19 was able to inhibit the growth of all pathogenic bacteria in this study except for *K. pneumoniae*, while *B. subtilis* HTI-23 could inhibit four out of six pathogenic bacteria. The remaining strains exhibited remarkable but lower antagonistic effects in comparison to *B. amyloliquefaciens* HTI-19 and *B. subtilis* HTI-23.

### 3.5. Tolerance to Acidic Conditions and Bile Salts

The effect of simulated gastrointestinal conditions on the viability of *B. amyloliquefaciens* HTI-19 and *B. subtilis* HTI-23 in comparison to *L. rhamnosus* GG is presented in Table 3. After exposure to acidic conditions (pH 2.0) and 0.3% bile salt solution for 3 h, the survival rates of *B. amyloliquefaciens* HTI-19 and *B. subtilis* HTI-23 were found to be >85%. In addition, both isolates exhibited significantly high survival rates in 0.3% bile salt solution compared to *L. rhamnosus* GG. 

### 3.6. Cell Adhesion Activity of Bacillus Species 

Autoaggregation is a probiotic characteristic that pertains to the entrapment of bacteria in an aggregated form, which allows for the stability of microbial strains in the gastrointestinal tract (GIT), resulting from lesser exposure to inhospitable intestinal conditions [35]. After 24 h of incubation, *B. amyloliquefaciens* HTI-19 and *B. subtilis* HTI-23 showed autoaggregation abilities of 84.13% and 57.51%, respectively (Table 4). Interestingly, the autoaggregation and hydrophobicity percentage of both *Bacillus* species showed no significant difference from *L. rhamnosus* GG. 

### 3.7. Antibiotic Susceptibility and Hemolytic Activity

The antibiotic susceptibility of the three probiotic *Bacillus* strains was tested using two groups of antibiotics categorized by their mechanisms. The two groups were cell wall inhibitors, including ampicillin, ciprofloxacin, kanamycin, streptomycin, and vancomycin. Protein synthesis inhibitors included are chloramphenicol, erythromycin, gentamicin, tetracycline, and teicoplanin. Antibiotics that could perform both mechanisms of action depending on the concentration and susceptibility of the bacteria like rifampicin were also included [36]. *B. amyloliquefaciens* HTI-19 and *B. subtilis* HTI-23 were susceptible to all antibiotics with different mechanisms of action, while *L. rhamnosus* GG was observed to be resistant to teicoplanin and vancomycin. Meanwhile, *B. amyloliquefaciens* HTI-19 showed α-hemolytic activity, while *B. subtilis* HTI-23 exhibited γ-hemolytic activity on a blood agar plate (Table 5).

## 4. Discussion

The amount of aerobic bacteria detected in the fresh honey of stingless bees could be considered relatively low, with a mean value of 1.7 x 10^2^ CFU/g, compared to its other byproducts, such as beebread and propolis (1.83 × 10^6^ CFU/g) [12,37]. This is supported by the results from a previous study, as the presence of aerobic bacteria in honey was also detected in the range of 5.7 × 10^0^ to 52.8 × 10^4^ CFU/g [38]. The reason for this is that most bacteria are not able to multiply in honey due to the physicochemical properties of honey itself, such as high osmolarity, high sugar concentration, low pH, and the presence of many agents, including hydrogen peroxide and phytochemicals [13,39]. These conditions provide a stressful environment for bacteria, thus preventing the growth or even survival of different types of bacteria in honey. Therefore, a high number of aerobic bacteria could indicate contamination during processing, handling, or storing.

In a previous study by Esawy et al. [8], the strains isolated from honeybees were rod-shaped, Gram-positive, motile, and spore-forming. All of the isolates were moderately thermophilic and were preliminary identified as *Bacillus* spp. The results are in agreement with the results obtained in our study, where most of the bacterial isolates were also Gram-positive and rod-shaped. Potential probiotic species, *B. amyloliquefaciens* and *B. subtilis*, were also isolated in this study. Previously, both *Bacillus* species had been isolated from the gut and honey of *Apis mellifera* [40]. Probiotic bacteria were commonly selected from the Gram-positive bacteria, as the cell surface structures of Gram-positive microbes can ensure effective bacterial adhesion to the intestinal cell wall [41]. This characteristic is really important to ensure the successful colonization of the host. 

A detailed analysis of the 16S rRNA gene sequences of the isolates exhibited significant diversity even in the case where bacteria were isolated from the same species of stingless bee. Environmental factors such as nectar, water, and pollen might be responsible for the diversity of the strains. A recent study found that the highest microbial diversity was found in multifloral honey [13]. Recently, the presence of *B. altitudinis* in stingless bee honey, *H. itama*, was reported for the first time [12]. *B. altitudinis* had been previously found in *Apis mellifera* honey together with other *Bacillus* isolates, namely *B. licheniformis, B. safensis, B. zhangzhouensis, and B. xiamenensis* [13]. This showed that *B. altitudinis* and *B. pumilus* have a niche in both honey samples of *H. itama* and *A. mellifera*. Interestingly, this species has been identified as one of the starter culture strains in rice wine [42]. Thus, the presence of these species in honey might suggest the roles of *B. altitudinis* in the fermentation of both *A. mellifera* and *H. itama* honeys.

*B. amyloliquefaciens* HTI-19 and *B. subtilis* HTI-23, which are associated with fermentation products, were successfully isolated from stingless bee honey. These species, together with *B. methylotrophicus, B. safensis*, and *B. vallismortis*, have been previously detected in *A. mellifera* honey, Korean traditional soy sauce, and the fermented seed condiment *Kantong* [8,43,44]. In fact, an assessment of cultivable microorganisms in honey has reported *B. amyloliquefaciens* to be the most prevalent strain among 13 species isolated from 38 honeys [45]. The 16S rRNA gene sequence of the *B. subtilis* HTI-23 isolate exhibited 99% sequence similarity to the *B. subtilis* strain BSFLG01 isolated from the black soldier fly larval gut, which is known as an invading species that has caused the mass infestation of domesticated stingless bees in Malaysia [46]. Hence, it was assumed that *B. subtilis* is a natural inhabitant in the honey.

Although it appears that there was no correlation between the microbial diversity and the geographical origin, the distinction of *Bacillus* strains found in the *H. itama* honey may be explained by the uses of the tubular proboscis of the bees while collecting nectar from various floral sources [47]. During the feeding process, the external surfaces of the bee’s frontal organs are in close proximity to the nectar, and bacteria are then inoculated into the honey, which confirms the role of bees as bacterial vectors. Strains of *B. amyloliquefaciens* ssp. *plantarum* and *B. methylotrophicus* that have plant growth-promoting abilities are frequently isolated from plant material and/or soil [48]. However, bees might contribute to the presence of these bacteria strains in stingless bee honey during the pollination of different plants. Hence, it was hypothesized that *Bacillus* strains isolated from *H. itama* honey might come from floral sources, transferred by the *H. itama* bee during its foraging flight [49].

Another purpose of this study was to select the bacteria that exhibited excellent antimicrobial activity against pathogenic bacteria. The successful selection of antimicrobial producers from honey has been reported by several different authors [13]. For example, Manhar et al. [50] reported that *B. amyloliquefaciens* AMS1 inhibited the growth of *L. monocytogenes* and *K. pneumoniae*, but did not affect the growth of *B. cereus, Yersinia enterocolitica*, and *Salmonella entericatyphimurium*. In contrast with our study, the *B. amyloliquefaciens* strain HTI-19 exhibited a wider antimicrobial spectrum against pathogens, as it can inhibit the growth of *S. thyphimurium* and *B. cereus.* Many attempts were have been made to prevent the growth of *B. cereus* in food products, because *B. cereus* is known to be one of the major threats to food safety. Further characterization of *B. amyloliquefaciens* HTI-19 will be particularly helpful in food industries. It has been reported that *B. amyloliquefaciens* strains were able to inhibit the growth of a variety of fungal pathogens because of their ability to produce a vast array of antibiotics, such as bacillomycin, zwittermicin, bacilysin, difficidin, and fengycin [51,52].

In addition, the growth of *S. aureus* was successfully inhibited by most of the *Bacillus* strains in this study. Since this study used cell-free supernatant for the antimicrobial activity assay, the potential antimicrobial metabolites produced were bacteriocin, hydrogen peroxide, and lactic and propionic acid [53]. Despite a few *Bacillus* spp. being known as toxin producers, some of the *Bacillus* strains are already considered to be safe probiotic bacteria. These include *B. endophyticus, B. subtilis, B. amyloliquefaciens, B. pumilus*, and *B. licheniformis* [8,43,50]. *Bacillus subtilis* has also been shown to have a broad spectrum of antimicrobial activities over diverse pathogenic fungal and bacteria [54].

Tolerance to low-acidic gastric and bile-rich intestinal environments is one of the essential properties required for probiotic cultures in order to function effectively in the intestines, because such conditions provide a stressful environment for bacteria [55]. The results obtained after 3 h established the possibility that the strain can survive under acidic conditions that exist in the human gut (pH 2–5), as the transit time of the food along the human gut is a maximum of 3 h [10]. This result suggests that *B. amyloliquefaciens* species have high levels of survival in simulated gastric juices (pH 2.0), as previously reported by Wang et al. [56]. Bile salts have been reported to inhibit bacterial growth by disrupting cell membranes. Some studies have observed that some *Bacillus* spp. are weakly tolerant or sensitive to bile salt concentrations [56]; however, the present results showed that the survival rates of *B. amyloliquefaciens* HTI-19 and *B. subtilis* HTI-23 in 0.3% bile salt solution were significantly higher (*p* < 0.05) than for *L. rhamnosus* GG. Tolerance to bile salt enables a probiotic strain to survive, grow, and exert itself during gastrointestinal transit [36].

The adherence ability of probiotic bacteria to intestinal epithelial cells involves various types of interactions, including hydrophobicity and autoaggregation [57]. The ability to adhere to epithelial cells and mucosal surfaces is considered to be a prerequisite for ideal probiotics. In this study, xylene was chosen as an apolar solvent because it reflects cell surface hydrophobicity and hydrophilicity [58]. As the results showed, both strains exhibited high hydrophobicity with xylene, indicating good bacterial adhesion to hydrocarbons. Patel et al. [25] have reported that the autoaggregation activity of *B. subtilis* DET6 is about 60%, which is in agreement with our study. These properties are crucial for probiotic cultures in colonizing epithelium cells in the digestive tract to prevent elimination by peristalses and to become functionally effective in intestinal balance [10]. Autoaggregation is also strongly correlated with cell adhesion to the digestive tract, which is responsible for the probiotic characteristics of bacteria [30]. The results showed that the two probiotic strains had high cell hydrophobicity and autoaggregation, indicating good cell adhesion ability.

The antibiotic susceptibility of probiotics should be measured for safety purposes. Antibiotic resistance gene transmission can occur due to transposons, plasmids, and bacterial gene mutations, leading to new antibiotic-resistant strains [59]. An antibiotic susceptibility test indicated that *B. amyloliquefaciens* HTI-19 and *B. subtilis* HTI-23 were sensitive to all antibiotics included in this study. Resistance to a given antibiotic can be inherent to a bacterial species or genus. In addition, γ-hemolysis and α-hemolysis are considered to be safe, and β-hemolysis is considered to be harmful [60] as β-hemolysis is an indication that bacteria contain cytotoxic phospholipases [61].

## 5. Conclusions

The results of the present research demonstrated that honey of different geographical origins in Malaysia can be considered as a reservoir of bacteria with antimicrobial activities, with potential for use as probiotic cultures. Interestingly, *B. amyloliquefaciens* HTI-19 not only showed a broad range of antimicrobial activities that could inhibit both Gram-positive and Gram-negative bacteria, but also was able to inhibit the growth of *B. cereus* and *S. thyphimurium*, which had not been inhibited previously by a different strain of *B. amyloliquefaciens* species in other studies. Two *Bacillus* strains (*B. amyloliquefaciens* HTI-19 and *B. subtilis* HTI-23) that were isolated from stingless bee honey possess great potential as probiotics for human and animal use and as fermentation starter cultures. This was supported by positive probiotic characteristics such as high survivability in the artificial modified digestive tract system, wide antimicrobial spectra, and safety confidence with regard to antibiotic susceptibility and nonhemolytic activity. The current findings suggest that these strains may exhibit the ability to remain viable after exposure to stressful environments in the gastrointestinal tract of humans and animals, thus being able to be functionally effective in the intestine. As probiotic effects on certain noncommunicable diseases have proven to be strain-specific, further investigation into these isolates may lead to the discovery of new beneficial probiotic strains that can be used in the therapeutic field.

## Figures and Tables

**Table 1:** Preliminary selection of *Bacillus* strains isolated from stingless bee honey from different geographical locations. CFU: colony-forming unit.

Geographical Location	CFU/g	No. of Selected Isolates	Gram-Staining				Catalase Test		Tolerance to 7% NaCl
			Gram + ve	Gram − ve	Bacilli	Cocci	+ve	−ve	
Batang Benar, Negeri Sembilan	2.2 × 10^2^	17	17	-	17	-	All isolates exhibited positive results for catalase test		6
Segamat, Johor	2.4 × 10^2^	23	21	2	23	-			9
Seri Kembangan, Selangor	1.1 × 10^1^	3	3	-	3	-			2
Sematan, Sarawak	9.7 × 10^0^	4	3	1	3	1	3	2	2
Serdang, Selangor	3.7 × 10^2^	11	11	-	11	-	6	5	4
							TOTAL		23

**Table 2:** Antagonistic activities of *Bacillus* species against six different pathogenic bacteria.

Bacterial Isolates	Inhibition Zones against Pathogenic Bacteria, mm					
	*Staphlococcus aureus*	*Bacillus cereus*	*Salmonella thyphimurium*	*Escheria coli*	*Klebsiella pneumonia*	*Pseudomonas aeruginosa*
*B. pumilus* HTI-1	+++	NI	NI	NI	NI	NI
*B. pumilus* HTI-2	+++	NI	NI	NI	NI	NI
*B. pumilus* HTI-3	NI	NI	NI	NI	NI	NI
*B. pumilus* HTI-4	++	NI	NI	NI	+	NI
*B. pumilus* HTI-5	++	NI	NI	NI	NI	NI
*B. pumilus* HTI-6	+++	NI	NI	NI	NI	NI
*B. pumilus* HTI-7	+++	++	NI	NI	++	NI
*B. pumilus* HTI-8	+++	NI	NI	NI	NI	NI
*B. altitudinis* HTI-11	NI	NI	NI	NI	NI	++
*B. altitudinis* HTI-14	+++	NI	NI	NI	NI	NI
*B. altitudinis* HTI-15	+++	NI	NI	NI	NI	NI
*B. megaterium* HTI-16	NI	NI	NI	NI	NI	NI
*B. megaterium* HTI-17	NI	NI	NI	NI	NI	NI
*B. megaterium* HTI-18	NI	NI	NI	NI	NI	NI
*B. amyloliquefaciens* HTI-19	+++	++	++	++	NI	+
*B. amyloliquefaciens* HTI-20	+++	NI	NI	+++	NI	NI
*B. aryabhattai* HTI-21	NI	NI	NI	NI	NI	NI
*B. aryabhattai* HTI-22	NI	NI	NI	NI	NI	NI
*B. subtilis* HTI-23	+	NI	+++	NI	++	++
*L. rhamnosus* GG	+++	+++	+++	+++	++	+++
Tetracycline(20ug/µl)	+++	+++	+++	+++	+++	+++

Note: clear zone around well; +: 1–3 mm; ++: 3–5 mm; +++: >5 mm; NI: no inhibition zone was detected.

**Table 3:** The survival of selected probiotic *Bacillus* isolates in simulated gastrointestinal conditions.

Isolates	Survival Rates, %	
	Acid Tolerance	Bile Tolerance
	pH 2.0	0.3%
*B. amyloliquefaciens* HTI-19	86.56 ^a^	129.10 ^a^
*B. subtilis* HTI-23	86.72 ^a^	140.50 ^b^
*L. rhamnosus* GG	97.46 ^b^	106.76 ^c^

^a^–^c^: Different superscript letters in the same column indicate statistical differences in each strain at the level of *p* < 0.05 as measured by Tukey’s test. All the results were obtained after 3 h, and the values are represented as mean SDs of three independent replicates.

**Table 4:** Cell adhesion activity of selected probiotic species.

Isolates	Autoaggregation (%)	Hydrophobicity (%)
*B. amyloliquefaciens* HTI-19	84.13 ^a^	53.64 ^a^
*B. subtilis* HTI-23	57.51 ^b^	60.82 ^a^
*L. rhamnosus* GG	69.99 ^ab^	61.04 ^a^

^a,b^: Different superscript letters in the same column indicate statistical differences in each strain at the level of *p* < 0.05 as measured by Tukey’s test. All the values are represented as mean SDs of three independent replicates.

**Table 5:** Antibiotic susceptibility of *Bacillus* isolates to antibiotics. Antibiotics: AMP10 (ampicillin, 10 μg); C30 (chloramphenicol, 30 μg); CIP5 (ciprofloxacin, 15 μg); E15 (erythromycin, 15 μg); CN10 (gentamicin, 10 μg); K30 (kanamycin, 30 μg); TE30 (tetracycline, 30 μg); TEC30 (teicoplanin, 30 μg); VA30 (vancomycin, 30 μg); RD30 (rifampicin, 30 μg); and S10 (streptomycin, 10 μg). R: resistant; S: susceptible.

Isolates	Susceptibility to Antibiotics											Hemolytic Activity
	AMP10	C30	CIP5	E15	CN10	K30	TE30	TEC30	VA30	RD30	S10	
*B. amyloliquefaciens* HTI-19	S	S	S	S	S	S	S	S	S	S	S	α-hemolytic
*B. subtilis* HTI-23	S	S	S	S	S	S	S	S	S	S	S	γ-hemolytic
*L. rhamnosus* GG	S	S	S	S	S	S	S	R	R	S	S	γ-hemolytic
